# Evolution of MHC class I genes in the European badger (*Meles meles*)

**DOI:** 10.1002/ece3.285

**Published:** 2012-07

**Authors:** Yung Wa Sin, Hannah L Dugdale, Chris Newman, David W Macdonald, Terry Burke

**Affiliations:** 1 Wildlife Conservation Research Unit, Department of Zoology, University of Oxford, Recanati-Kaplan CentreTubney House, Abingdon Road, Tubney, Abingdon, Oxfordshire OX13 5QL, United Kingdom; 2 NERC Biomolecular Analysis Facility, Department of Animal and Plant Sciences, University of SheffieldWestern Bank, Sheffield, South Yorkshire, S10 2TN, United Kingdom; 3 Behavioural Ecology and Self-Organization, University of GroningenP.O. Box 11103, 9700 CC Groningen, The Netherlands; 4 Theoretical Biology, University of GroningenP.O. Box 11103, 9700 CC Groningen, The Netherlands

**Keywords:** Balancing selection, birth-and-death evolution, concerted evolution, major histocompatibility complex, orthology, trans-species polymorphism

## Abstract

The major histocompatibility complex (MHC) plays a central role in the adaptive immune system and provides a good model with which to understand the evolutionary processes underlying functional genes. Trans-species polymorphism and orthology are both commonly found in MHC genes; however, mammalian MHC class I genes tend to cluster by species. Concerted evolution has the potential to homogenize different loci, whereas birth-and-death evolution can lead to the loss of orthologs; both processes result in monophyletic groups within species. Studies investigating the evolution of MHC class I genes have been biased toward a few particular taxa and model species. We present the first study of MHC class I genes in a species from the superfamily Musteloidea. The European badger (*Meles meles*) exhibits moderate variation in MHC class I sequences when compared to other carnivores. We identified seven putatively functional sequences and nine pseudogenes from genomic (gDNA) and complementary (cDNA) DNA, signifying at least two functional class I loci. We found evidence for separate evolutionary histories of the α1 and α2/α3 domains. In the α1 domain, several sequences from different species were more closely related to each other than to sequences from the same species, resembling orthology or trans-species polymorphism. Balancing selection and probable recombination maintain genetic diversity in the α1 domain, evidenced by the detection of positive selection and a recombination event. By comparison, two recombination breakpoints indicate that the α2/α3 domains have most likely undergone concerted evolution, where recombination has homogenized the α2/α3 domains between genes, leading to species-specific clusters of sequences. Our findings highlight the importance of analyzing MHC domains separately.

## Introduction

The major histocompatibility complex (MHC) is of particular importance to the study of evolutionary genetics owing to its pattern of molecular evolution. MHC is a diverse gene family that plays a crucial role in the vertebrate adaptive immune system and in autoimmunity. Cell surface glycoproteins, encoded by the MHC genes, are vital in both humoral and cell-mediated immune responses, as they bind and present antigens to T cells and trigger an immune cascade (Swain 1983). MHC genes are classified into groups including class I and class II. MHC class II molecules principally bind peptides from the extracellular environment and are only expressed on antigen-presenting cells, such as B cells and macrophages (Hughes and Yeager 1998). MHC class I genes comprise classical (class Ia) and nonclassical (class Ib) loci that differ in polymorphism, structure, function, and expression pattern (Parham and Ohta 1996; Rodgers and Cook 2005). MHC class Ia molecules are responsible primarily for intracellular antigen binding and are expressed on the surface of all nucleated somatic cells (Bjorkman and Parham 1990). Because of this crucial role in the immune system, MHC genes are under constant selective pressures due to challenges from parasites and pathogens (Jeffrey and Bangham 2000; Piertney and Oliver 2006). This arms race between pathogens and hosts is posited to be the driving force for the extreme diversity in MHC genes (e.g., class Ia genes such as HLA-A, HLA-B, and HLA-C in humans; Piertney and Oliver 2006).

The high degree of diversity among MHC alleles arises from the diverse exons that encode the domains forming the antigen-binding site (ABS; Hughes and Yeager 1998). The nucleotide diversity within the MHC genes has been attributed to balancing selection (Bergström and Gyllensten 1995), such as overdominance and frequency-dependent selection, which act to maintain large numbers of alleles in populations. The persistence of ancestral allelic diversity over long periods of time, relative to neutral genetic variation (Richman 2000), is enhanced substantially by balancing selection, which leads to high levels of allelic diversity within species (Hughes and Yeager 1998; Penn et al. 2002). Some mammalian MHC allelic lineages are more than a million years old and are maintained after speciation (Figueroa et al. 1988). Phylogenetic reconstructions therefore reveal trans-species polymorphism (Klein 1987; Klein et al. 1998, 2007), where alleles between species are more closely related (or even identical) than alleles within species. Phylogenetic reconstructions can also reveal orthologous relationships, by which sequences group by gene rather than by species, forming orthologous gene clusters (Nei et al. 1997; Nei and Rooney 2005). In orthologous clusters, the diveregnce pattern of genes reflects species phylogeny (when trans-species polymorhism does not also occur) because genes have diverged from a common ancestor due to a speciation event (Fitch 2000).

In contrast to class II genes, which usually exhibit orthology and trans-species polymorphism in mammals, class I genes tend to form monophyletic groups within species and therefore it is difficult to establish orthologous relationships among closely related mammalian species (Takahashi et al. 2000). One plausible explanation is that class I loci undergo a faster rate of birth-and-death evolution than do class II loci (Nei et al. 1997; Vogel et al. 1999; Takahashi et al. 2000; Piontkivska and Nei 2003), whereby new genes are created by gene duplication and some genes become nonfunctional through deleterious mutations, or become deleted from the genome. The orthologous relationship may therefore become lost due to birth-and-death evolution. The high divergence of class I genes between closely related species has also been posited to be due to frequent genetic exchange by recombination or gene conversion termed “concerted evolution” (Rada et al. 1990; Hess and Edwards 2002; Nei and Rooney 2005). This yields genes within a species that are more similar to each other than they are to the orthologs in closely related species; that is, intraspecific paralogs are more similar than interspecific orthologs. The homogenizing effect of recombination can mask orthology and duplication history and impede the reconstruction of the phylogeny. With higher rates of concerted evolution, divergence between orthologs increases, which makes the signal for orthology harder to detect. In the concerted evolution model, different loci are homogenized constantly by gene conversion (Rada et al. 1990; Nei and Rooney 2005). This tends to eliminate variation and generates intraspecific similarity while reducing interspecific similarity, thus facilitating diversification. Exchange of sequence segments by gene conversion, and recombination, plays a prominent role in the evolution of the MHC genes (Parham and Ohta 1996; Jakobsen et al. 1998; Richman et al. 2003). In addition to its homogenization effect, it has been proposed that recombination creates and maintains diversity in the MHC genes (Holmes and Parham 1985; Jakobsen et al. 1998). The consequent phylogenetic inconsistencies between different regions of the same gene, due to concerted evolution, have been observed in the MHC of many species (e.g., Shum et al. 2001; Bos and Waldman 2006; Burri et al. 2008), including humans (Houlden et al. 1996; Jakobsen et al. 1998; Bos and Waldman 2006).

To date, the domestic dog, *Canis lupus familiaris,* has provided the model for MHC research in carnivores. In the dog leukocyte antigen (DLA; Kennedy et al. 2001; Wagner 2003), one class Ia gene is present, with three class Ib genes and two pseudogenes. This class Ia gene is highly polymorphic, with more than 50 alleles identified (Wagner 2003). Studies of the MHC class I genes in many carnivores, such as those in the superfamily Musteloidea, are however lacking. Here, we characterize the MHC class I genes of the European badger (*Meles meles*). *Meles meles* is well suited to investigate how MHC selection and conferred immunological advantages (e.g., pathogen and parasite resistance) are regulated by mate choice in the wild. *Meles meles* has a long mating season (Buesching et al. 2009), delayed implantation (Thom et al. 2004), putative superfoetation (Yamaguchi et al. 2006), and a sensory predisposition toward olfaction (Buesching et al. 2002). In high-density populations *M. meles* has a polygynandrous mating system (Dugdale et al. 2007, 2011) with high levels of extra-group paternity (Dugdale et al. 2007) and low fecundity (Macdonald et al. 2009). Additionally, it has been the subject of a diverse range of endoparasitic disease studies (Macdonald et al. 1999; Anwar et al. 2000, 2006; Newman et al. 2001; Rosalino et al. 2006; Nouvellet et al. 2010; Lizundia et al. 2011). In particular, *M. meles* is a wildlife reservoir of *Mycobacterium bovis* (Delahay et al. 2001; Mathews et al. 2006; Riordan et al. 2011); an intracellular bacteria that is the cause of bovine tuberculosis (bTB) in cattle and wildlife. MHC class I-dependent immunity is known to play an important role in the eradication of *M. bovis* in mice (Ladel et al. 1995). *Meles meles* with different MHC genotypes, or the presence/absence of certain MHC alleles, may therefore have differential susceptibilities to *M. bovis*, which could contribute to genetic-based bTB control strategies in badgers.

In this study we: (1) characterized the MHC class I genes of *M. meles* from a high-density population and tested for evidence of selection and recombination; (2) identified the transcription pattern by comparing genomic DNA (gDNA) and complementary DNA (cDNA) sequences from whole blood samples, which is important as MHC genes identified using gDNA may be nonfunctional; and (3) performed phylogenetic analyses to investigate whether *M. meles* sequences belong to monophyletic groups, or whether sequences transcend species boundaries. Characterization of MHC class I genes in *M. meles* will clarify whether these have a more rapid turnover rate than class II loci (Sin et al. 2012) and facilitate elucidation of the underlying evolutionary processes within different regions. Moreover, the development of MHC markers will facilitate studies on the relationship between genetics and disease in this controversial animal, as well as other closely related species.

## Materials and Methods

### Sample collection and nucleic acid isolation

Blood samples were collected from 11 badgers that resided in eight different social groups (Sin et al. 2012) in Wytham Woods, Oxfordshire, UK (global positioning system reference 51°46′26N, 1°19′19W). All trapping and handling protocols are detailed in Macdonald and Newman (2002). These protocols were subject to ethical review and were performed under Natural England Licence (currently 20104655) and UK Home Office Licence (PPL 30/2835). Approximately 3 mL of blood was taken by jugular venipuncture and collected in a vacutainer containing EDTA. Samples were stored at −20°C until DNA isolation was performed. gDNA was isolated using the GFX Genomic Blood DNA Purification Kit (Amersham Biosciences, Little Chalfont, UK), following the scalable method in the manufacturer's protocol. In order to validate whether the identified alleles were transcribed, a 500-μL blood sample, from each of the 11 individuals, was also transferred into RNAprotect Animal Blood Tubes (Qiagen, Hilden, Germany) and stored immediately at −20°C for less than a month before RNA isolation. Total cellular RNA was isolated from each blood sample using an RNeasy Protect Animal Blood Kit (Qiagen). Methods of cDNA synthesis are detailed in Sin et al. (2012).

### Primer design and polymerase chain reaction (PCR) amplification

**Figure 1:**
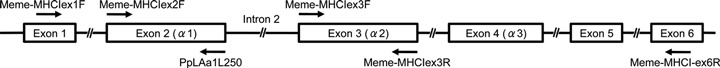
Schematic representation of the positions of the primers used for amplification of MHC class I sequences from cDNA/gDNA. α1−3 domains are labeled at the exons encoding them.

MHC class I molecules are heterodimers, that consist of an α chain and a β_2_-microglobulin (β_2_m) molecule. The α chain is composed of three extracellular domains α1, α2, and α3 (encoded by exon 2, 3, and 4, respectively), a transmembrane domain (exon 5), and a cytoplasmic domain (exon 6; Fig. 1; Bjorkman and Parham 1990). The α1 and α2 domains are α chain regions that comprise specific sites forming the ABS.

To amplify the class I genes from *M. meles*, we tested published primers used successfully in other carnivores (Zhong et al. 1998; Aldridge et al. 2006). Oligonucleotide primers, which recognize highly conserved regions of the MHC class I genes, were also designed using OligoAnalyzer 3.1 (Owczarzy et al. 2008), based on alignments with GenBank's nucleotide sequences from domestic dog (*Canis lupus familiaris*; AF218297, AF218299, AF218301, AF218303, DQ056267, DQ056268, and M32283), harbour seal (*Phoca vitulina*; U88874), domestic cat (*Felis catus*; M26318, U07670, U07672 and U07674), horse (*Equus caballus*; NM001123381), and human (*Homo sapiens*; AF287959, U03907, NM002117, and NM002127).

Using these primers on 10–30 ng of cDNA/gDNA, PCR amplification was performed in a 20-μL reaction mix that also contained 0.5 μM of each primer (Table 1), 200 μM of each dNTP, 1× PCR buffer (containing MgCl_2_; Qiagen), and 2 units of HotStarTaq (Qiagen). The PCR cycle began with incubation at 94°C for 15 min, followed by 35 incubation cycles at 94°C for 30 sec, annealing temperature (Table 1) for 30 sec, and 72°C for 60–90 sec according to amplicon length (60 sec for amplification of exon 2 or exon 3 only), ending with an extension step at 72°C for 10 min. The PCR products were electrophoresed on a 1.5% agarose gel and visualized using ultraviolet light and ethidium bromide staining. A 100-bp DNA ladder (New England Biolabs, Herts, UK) was used to size the DNA fragments. Bands of expected size were excised from the gel and purified using QIAquick Gel Extraction Kits (Qiagen). PCR products that gave rise to relatively bright bands of the expected size were cloned and sequenced.

**Table 1:** MHC class I-specific primers for *Meles meles.*

Primer name	Primer sequence	Product size (bp): region amplified	*T_a_* (°C)	Source reference
F: Meme-MHCIex1F	GGCCCTGGCCGTGACC	1012 bp; exon 1–exon 6	59	This study
R: Meme-MHCI-ex6R	ATCAGAGCCCTGGGCACTGTC			This study
F: Meme-MHCIex2F	GGCTCCCACTCCCTGAGG	543 bp: exon 2–exon 3 on cDNA	59	This study
R: Meme-MHCIex3R	GCGCAGCAGCGACTCCTT	731–784 bp: exon 2–exon 3 on gDNA		This study
R: PpLAa1L250	GGCCTCGCTCTGGTTGTAG	270 bp; exon 2	55	Aldridge et al. (2006)
F: Meme-MHCIex3F	GGGTCTCACACCATCCAG	(Pairs with Meme-MHCIex3R) 273 bp; exon 3	59	This study

F, forward; R, reverse; bp, base pair; *T_a_*, annealing temperature.

### Cloning and DNA sequencing

Purified PCR fragments were cloned using the pGEM-T Easy Vector Systems (Promega, Madison, WI). The cloning and sequencing procedure is detailed in Sin et al. (2012). Between nine and 72 clones were sequenced for each individual. Identical sequences were derived from a minimum of two badgers or from independent PCR reactions from the same individual, in compliance with DLA nomenclature rules (Kennedy et al. 1999). Single unique sequences (possible chimeras) were excluded. Nucleotide sequences were analyzed using CodonCode Aligner 3.7.1 (CodonCode Corporation, Dedham, MA) and were compared with known MHC class I sequences using the NCBI BLAST program (Altschul et al. 1990). The DNA sequences from *M. meles* were assigned the GenBank accession numbers JQ425427−JQ425447.

## Data Analyses

### Selection and recombination

Selection at the amino acid level was measured as the rates of nonsynonymous (*d*_N_) and synonymous (*d*_S_) substitutions per codon site, estimated in DnaSP 4.0 (Rozas et al. 2003) and MEGA 4 (Tamura et al. 2007) according to the method of Nei and Gojobori (1986), with Jukes and Cantor (1969) correction. Standard errors were derived from 1000 bootstrap replicates. Synonymous and nonsynonymous substitutions were calculated separately for the ABS and non-ABS, as determined by Bjorkman et al. (1987). CODEML in PAML 4.4b (Yang 2007) was used to check for positively selected sites (PSS) in the α1 and α2 domains, which are indicated where the ratio ω (nonsynonymous/synonymous substitution rate ratio, *d*_N_/*d*_S_) exceeds 1, meaning that nucleotide mutations that alter the amino acid sequence of a protein occur more frequently than nucleotide mutations that do not alter amino acids. Thus, ω >1 is indicative of positive selection whereby beneficial amino acid changes are fixed. Codon-based likelihood analysis was used to test for evidence of positive selection, using several models: M1a (nearly neutral), M2a (positive selection), M7 (beta), and M8 (beta and ω). The assumptions of these models are detailed in Yang et al. (2000, 2005). Two null models of neutral evolution (M1a and M7) were applied and compared against their nested models, which allow stringent testing for positive selection (M2a and M8, respectively, which assume a different distribution of mutations; Anisimova et al. 2001, 2002). We determined whether the alternative models (M2a and M8) provided a significantly improved fit, versus their null models (M1a and M7, respectively), using a likelihood ratio test (LRT), which compares twice the difference of the log likelihood ratios (2ΔInL) to a *χ^2^* distribution. To identify codons under positive selection (posterior probabilities >0.95), through comparisons of M1a versus M2a and M7 versus M8, CODEML was used to calculate the Bayes Empirical Bayes (BEB) posterior probabilities (Yang et al. 2005) at each codon. Different domains were analyzed separately, as their evolutionary histories could be different due to recombination events in the intervening intron.

Recombination analyses were performed on the nucleotide alignment spanning exon 2, intron 2, and exon 3 using RDP3 alpha 44 (Martin et al. 2010). Four methods, that is, RDP (Martin and Rybicki 2000), GENECONV (Padidam et al. 1999), MaxChi (Smith 1992), and Bootscan (Martin et al. 2005), were applied in the first run to detect recombination events. Default settings were applied with a maximum *P*-value of 0.05, applying Bonferroni correction for multiple comparisons. Any recombination signals, which were detected by at least three methods, were then rechecked with all available methods (Martin et al. 2010). Any putative recombination events detected were verified further by examination of MaxChi plots and matrices and Neighbor-Joining trees from the inferred fragments, to assess recombinant designation and breakpoint placement. Only the recombination events that were confirmed after these procedures were considered significant. The effects of recombination and gene conversion on sequence evolution are similar in small sequence fragments, therefore we did not differentiate between them, and we refer to them as recombination sensu lato hereafter (Richman et al. 2003; Burri et al. 2008).

## Phylogenetic Analyses

Phylogenetic analyses were performed on the consensus alignments of *M. meles* MHC class I exon 2, exon 3, and exon 4 sequences, against sequences from other species available in GenBank (e.g., *C. lupus familiaris*[accession number: M32283, NM001014378, NM001014379, NM001014767, NM001020810, U55029], *P. vitulina*[PVU88874], *F. catus*[U07672, U07674], *E. caballus*[LOC100056062], *H. sapiens*[AF287959, NM002117.4, NM002127.5, U03907], *Ailuropoda melanoleuca*[EU162658, EU162659], *Leopardus pardalis*[U07678], *Acinonyx jubatus*[U07666], *Bos taurus*[AB245424], and *Monachus schauinslandi*[Aldridge et al. 2006]). Tree reconstruction was performed separately on three domains, in order to maximize the detection of different evolutionary histories due to genetic exchange. Domain borders were assigned according to Koller and Orr (1985).

Phylogenetic networks allow visualization of reticulate phylogenetic signals (Huson and Bryant 2006) whereas phylogenetics trees may poorly describe complex evolutionary scenarios; thus, phylogenetic networks provide an effective way of evaluating evolutionary relationships involving gene duplication and recombination. We used the Neighbor-Net algorithm in SplitsTree 4.12.3 (Huson and Bryant 2006) to analyze the phylogenetic relationships for exon 2, exon 3, and intron 2. We constructed a Neighbor-Net network based on uncorrected *p*-distances and conducted 1000 bootstrap replicates to estimate their support.

Bayesian phylogenetic inference was performed using MrBayes 3.1.2 (Ronquist and Huelsenbeck 2003) to analyze the phylogenetic relationship of exon 4. We used the web-based application FindModel (http://www.hiv.lanl.gov/content/sequence/findmodel/findmodel.html) to ascertain the best-fit model of nucleotide substitution, which was identified as the Hasegawa–Kishino–Yano plus gamma model (HKY+Γ). A Markov chain Monte Carlo (MCMC) search was initiated with random trees and run for 3,000,000 generations, sampling every 100 generations. Data were partitioned by gene codon positions. Two separate analyses and four independent chains were executed. Convergence was indicated when the average standard deviation of split frequencies was less than 0.01 (Ronquist et al. 2005). We also checked for convergence by plotting the likelihood scores against generations and discarded the first 25% of the generations as “burn-in.”

## Results

### Diversity and transcription of MHC class I sequences

**Figure 2:**
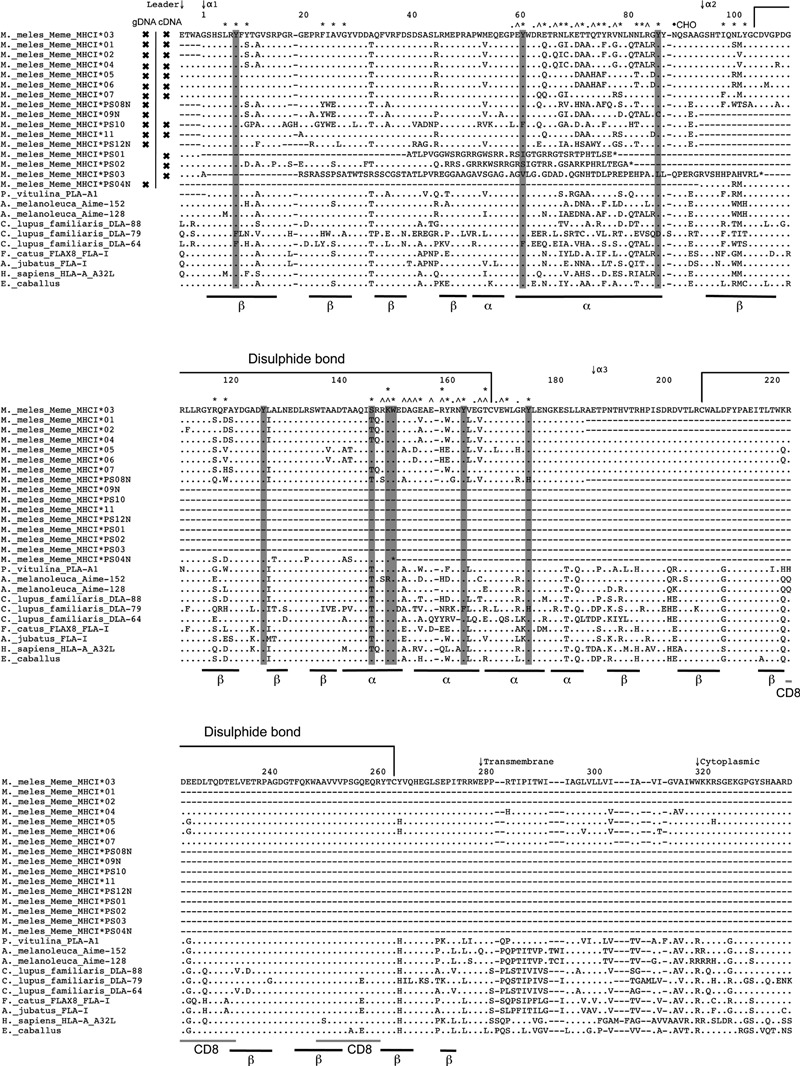
Amino acid sequence identity for class I sequences of *Meles meles* and seven other mammals (*Phoca vitulina*, *Ailuropoda melanoleuca*, *Canis lupus familiaris*, *Felis catus*, *Acinonyx jubatus*, *Equus caballus*, and *Homo sapiens*; GenBank accession numbers are provided in the Materials and Methods). The complete amino acid sequence of Meme-MHC I*03 is shown. *PS* signifies pseudogenes with a frameshift. *N* signifies the presence of genomic sequences, where no cDNA sequences were found. Single letters and dots within the alignment/sequence represent amino acids that are distinct from or identical to Meme-MHC I*03, respectively. Dashes (-) indicate missing sequences. Numbers above the sequence indicate the codon position. Arrows above the sequence label the beginning of a domain. Asterisks (*) indicate amino acid residues pointing toward the postulated antigen-binding site, which were defined according to Bjorkman et al. (1987). Carets (^∧^) indicate residues pointing up on an alpha-helix, postulated to interact with peptides and/or T-cell receptors (TCRs), and dots above the sequence (.) indicate residues on an alpha-helix that is pointing away from the antigen-binding site, postulated to interact with TCRs (Bjorkman et al. 1987). Conserved sites that bind the peptide N- and C-termini are marked with gray boxes. The location of the N-linked glycosylation site is at position 88 (ċCHO). Disulphide bonds formed between cysteine residues are shown with a line spanning the two cysteine residues. Residues that form the β-sheet or α-helix, and residues that influence the binding of the CD8 glycoprotein, are marked under the alignment. The columns on the left of the sequences indicate whether the sequences were found in gDNA and/or cDNA.

Sixteen different sequences were isolated from gDNA and cDNA, using the primers detailed in Table 1, Figure 1, and Table S1. A maximum of four putatively functional sequences was derived from a single individual, indicating the presence of at least two loci. Transcription analysis showed that nine of 16 sequences detected in the gDNA were also amplified from the cDNA, which was isolated from whole blood (Fig. 2; Table 2), while four sequences were detected only in the gDNA (Fig. 2).

**Table 2:** Presence of MHC class I sequences in genomic DNA (gDNA) and complementary DNA (cDNA) from 11 *Meles meles*. The total number of clones with different sequences found is given. Total number of clones: numbers out of brackets are clones of polymerase chain reaction (PCR) products from gDNA (numbers in brackets are clones of PCR products from cDNA)

	Individual												
		
Sequence	1	2	3	4	5	6^1^	7^1^	8^1^	9^2^	10^2^	11^2^	All	Total no. of clones
Meme-MHC I*01					X							X	3 (2)
Meme-MHC I*02				X								X	8 (5)
Meme-MHC I*03			X				X	X				X	3 (27)
Meme-MHC I*04	X	X	X	X	X	X	X					X	7 (72)
Meme-MHC I*05	X	X	X	X	X	X	X	X				X	88 (49)
Meme-MHC I*06	X							X				X	12 (14)
Meme-MHC I*07	X							X				X	5 (26)

X represents sequence from cDNA. Blank represents no detected sequence. Gray shading indicates sequence from gDNA.

1 gDNA was not sequenced.

2 cDNA was not sequenced.

**Figure 3:**
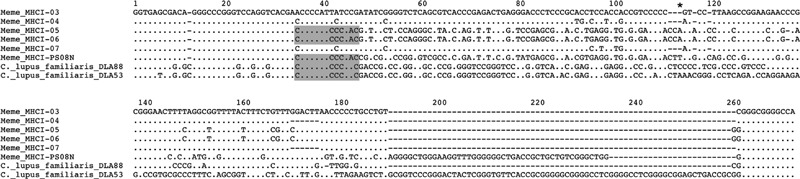
Nucleotide sequence identity for the MHC class I intron 2 of *Meles meles* clones and *C. lupus familiaris*. The GenBank accession numbers for sequences from *C. lupus familiaris* are NW876254.1 and U55029.1. The complete nucleotide sequence of Meme-MHC I*03 is shown; numbers above the sequence indicate the nucleotide position. *PS* signifies pseudogenes with a frameshift. *N* signifies the presence of genomic sequences, where no cDNA sequences were found. Single letters and dots (.) represent nucleotides that are distinct from or identical to Meme-MHC I*03, respectively. Dashes (-) indicate missing sequences. The degenerate 13-bp sequence motif (CCNCCNTNNCCNC) that is crucial in crossover events at human recombination hotspots (Myers et al. 2008) is marked with *gray boxes*. The nucleotide breakpoint for Meme-MHC I*05 and Meme-MHC I*06 is marked with an asterisk (*) above the alignment.

**Figure 4:**
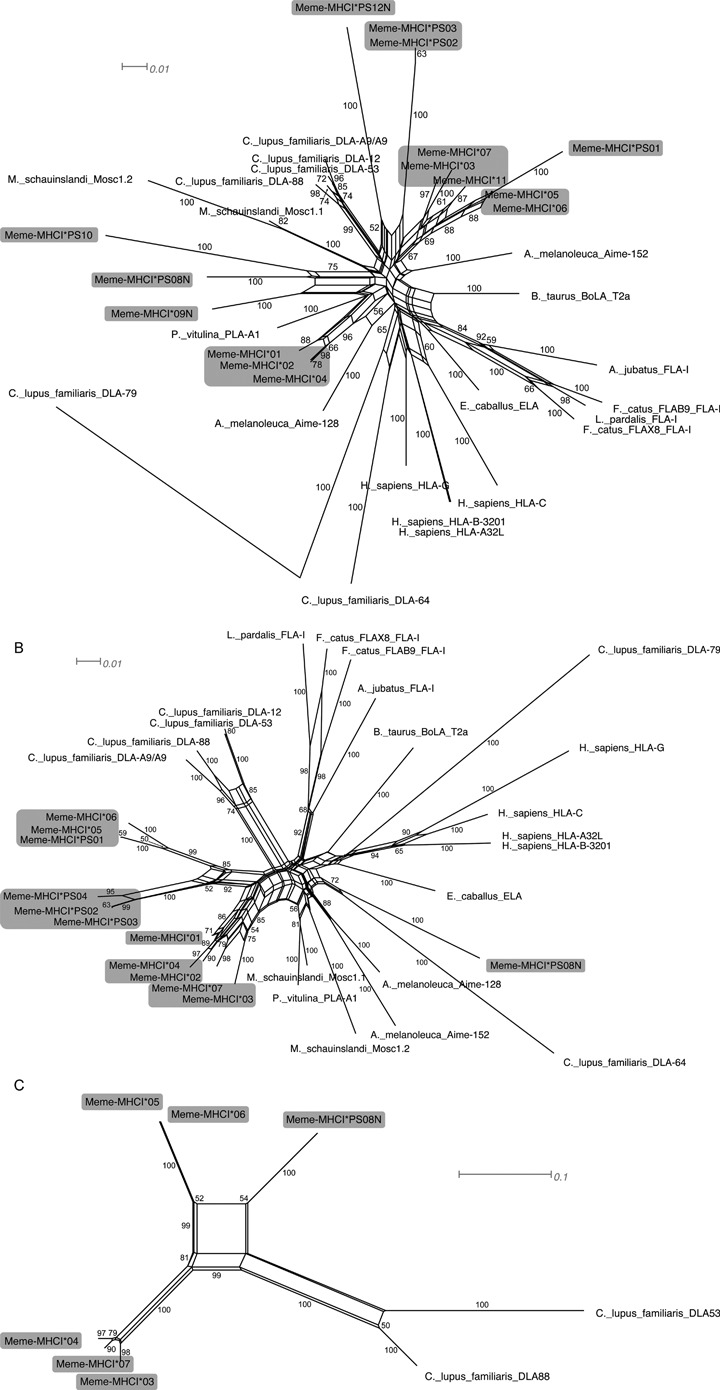
Neighbor-Net networks of MHC class I (A) exon 2/α1-domain, (B) exon 3/α2-domain, and (C) intron 2 sequences from *Meles meles* and other mammalian species including phocine, ursine, canine, feline, equine, and human (GenBank accession numbers are provided in the Materials and Methods). *Meles meles* class I sequences are marked with gray boxes. *PS* signifies pseudogenes with a frameshift. *N* signifies the presence of genomic sequences, where no cDNA sequences were found. Only bootstrap support values above 50 are shown; the central webbing indicates loops that have conflicting support for alternative branching events. Inclusion of 45 DLA-88 alleles (i.e., different major allelic types, Kennedy et al. 1999; 2001) formed a distinct clade comprising DLA-88, 12, 53, and A9, as shown in Figure 4; therefore, only one allele for DLA-88 is shown.

Pseudogene features (Fig. 2) were found in four sequences: nucleotide deletions caused a frameshift for Meme-MHC I*PS01, Meme-MHC I*PS02, Meme-MHC I*PS03, and Meme-MHC I*PS04N, which caused premature stop codons (Fig. 2; *PS* signifies pseudogenes with a frameshift, *N* signifies the presence of genomic sequences, where no cDNA sequences were found). Pseudogene Meme-MHC I*PS04N was detected only in gDNA, whereas Meme-MHC I*PS01, Meme-MHC I*PS02, and Meme-MHC I*PS03 were only detected in the cDNA, indicating the presence of a transcribed nonfunctional pseudogene (Mayer et al. 1993; Fernandez-Soria et al. 1998). The nucleotide deletion in Meme-MHC I*PS01 and Meme-MHC I*PS03 occurred at the 5′ primer annealing site for exon 2 amplification from gDNA; thus, no sequence was detected from gDNA. Nucleotide insertions or deletions were detected in two of four sequences that only amplified in the exon 2 region (Meme-MHC I*09N, Meme-MHC I*PS10, Meme-MHC I*11, and Meme-MHC I*PS12N; Fig. 2). The intron 2 of Meme-MHC I*PS08N is 45–56 bp longer than other putatively functional sequences; a longer intron was also found in a DLA pseudogene (DLA-53; Wagner 2003; Figs. 3 and 4C). Accordingly, the above sequences were regarded as pseudogenes and were not included in the nucleotide substitution calculations. Only sequences without pseudogene features, and with both exon 2 and exon 3 amplified from both gDNA and cDNA, were regarded as functional sequences and included in further analyses of nucleotide substitution and selection (i.e., Meme-MHC I*01−Meme-MHC I*07; Table 2; Fig. 2).

Of the seven putatively functional sequences, all were detected in both gDNA and cDNA (Table 2; Fig. 2). Meme-MHC I*05 was detected in all individuals. By contrast, Meme-MHC I*01 and Meme-MHC I*02 were identified and expressed in only one individual in this study (Table 2). One thousand one hundred and fifty-three individuals from the same population have been genotyped subsequently and these sequences have been identified in more individuals (330 for Meme-MHC I*01 and 132 for Meme-MHC I*02; Y. W. Sin, unpubl. data).

The majority of variable sites in the identified sequences were in exon 2 and exon 3, where most mutations represented a nonsynonymous nucleotide substitution (Table 3). As a consequence, there were more polymorphic amino acid residues among the α1 and α2 domains (Fig. 2; Table 3), which are involved in antigen binding. The average sequence divergence was 6.5% (SE = 0.62%); 7.0% (SE = 0.84%); and 1.0% (SE = 0.25%) in exon 2, exon 3, and exon 4, respectively. Two of the sequences, Meme-MHC I*02 and *04*, were highly similar, differing from each other at only one nucleotide position in exon 2 (Fig. 4A) and five positions in exon 3 (Fig. 4B). Meme-MHC I*03 was highly divergent (9.3−9.6%) from Meme-MHC I*01, *02* and *04*; Meme-MHC I*07 was highly divergent (9.3−9.6%) from Meme-MHC I*02 and *04*, in the exon 2 region. In the exon 3 region, Meme-MHC I*05 and *06* were highly divergent from the other five sequences (8.8−12.3%). The intron 2 region of Meme-MHC I*05 and *06* was identical (Figs. 3 and 4C) yet both of them were highly divergent from other putatively functional sequences (28.3−30.1%).

**Table 3:** Sequence polymorphism of MHC class I genes/molecules delineated by exon/domain (leader, α1, α2, α3, transmembrane, and cytoplasmic domains encoded by exon 1, 2, 3, 4, 5, and 6, respectively). The number of nucleotide and derived amino acid sequences isolated from 11 *Meles meles* were compared and the numbers of synonymous and nonsynonymous nucleotide substitutions and polymorphic amino acid residues are shown

Exons	1^1^	2	3	4	5	6^1^
Sequences for comparison	5	7	7	5	5	5
Variable sites	0	37	44	5	7	2
Mutations	0	39	47	5	7	2
Synonymous	0	7	14	2	1	1
Nonsynonymous	0	28	26	3	6	1
No. of amino acids	4	90	92	92	29	18
Polymorphic amino acid residues	0	23	24	3	6	1

1 Only part of the exon is included in this study.

Nine amino acid residues (three in the α1 domain and six in the α2 domain) are located at the two ends of the binding groove of the MHC molecule, where these residues bind the N- and C-termini of the presented peptide; these residues are highly conserved across vertebrates (Y7, Y59, Y84, Y123, T143, K146, W147, Y159, and Y171; Kaufman et al. 1994; see also Shum et al. 1999; Mesa et al. 2004). In *M. meles*, all of these residues except one were conserved (Fig. 2). Residue T143 is polymorphic in *M. meles* and four sequences have the common threonine (T), which is the same amino acid seen in six of seven species included in the sequence alignment (Fig. 2). The seventh species, *E. caballus*, has a serine (S) instead, which is the same as in the other three *M. meles* sequences.

The *M. meles* sequences also coded for other conserved residues, characteristic of class I molecules, which are important for the molecular structure and peptide binding. These include four cysteine (C) residues (positions 103, 167, 206, and 262; Fig. 2) that form the intradomain disulphide bonds, an N-linked glycosylation site in the α1 domain (position 88; Fig. 2); and a threonine (T) residue at position 136 (Fig. 2) that is critical for interactions with T-cell antigen processing (TAP) complexes and endogenous peptide loading (Peace-Brewer et al. 1996). Two regions in the α3 domain (position 222–232 and 248–259; Fig. 2), bearing many negatively charged residues, interact with the positively charged side chains of the CD8 glycoprotein on the T cells (Kaufman et al. 1994). Twenty of the 23 residues in these two regions are conserved in the *M. meles* sequences, except that glutamic acid (E224) replaces glycine (G) in Meme-MHC I*03, Meme-MHC I*04, and Meme-MHC I*07, leucine (L227) replaces glutamine (Q) in all sequences, and glutamine (Q256) replaces glutamic acid (E) in all sequences. The glutamine (Q229) residue (Fig. 2), which is important for CD8 accessory functions (Durairaj et al. 2003), is conserved here and in other mammalian, avian, and reptilian sequences (Kaufman et al. 1994).

### Selection and recombination

**Figure 5:**
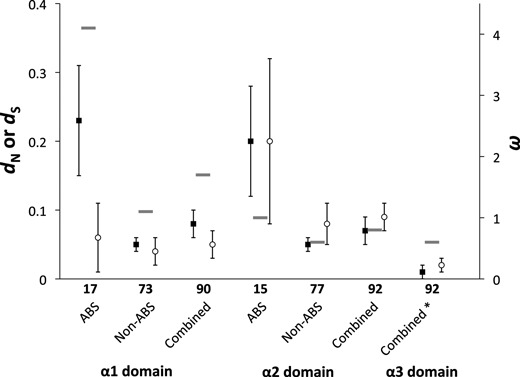
Rates (± standard error) of nonsynonymous (*d*_N_; solid marker) and synonymous (*d*_S_; open marker) substitutions and ratio of *d*_N_ to *d*_S_ (ω; gray bar) for antigen-binding site (ABS), non-ABS, and combined (ABS + non-ABS) at the three α domains of the *Meles meles* MHC class I loci. The number of codons for each region is given under the *x*-axis. Asterisk (*) indicates no ABS in α3 domain.

**Figure 6:**
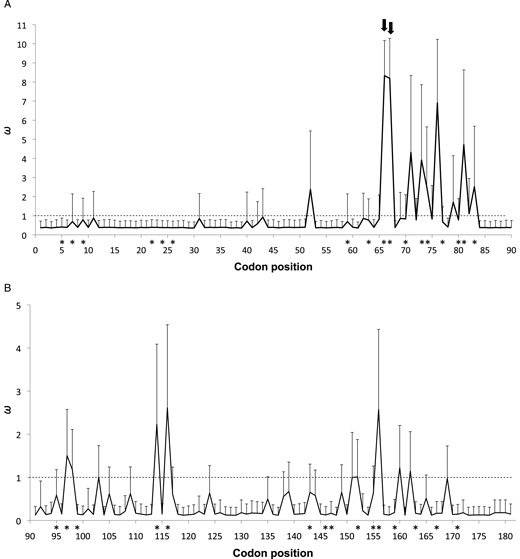
Mean ω at each codon position across (A) exon 2 and (B) exon 3 of *Meles meles* class I genes. Means of ω were calculated as the average of ω over the 11 site classes, weighted by the posterior probabilities under M8 computed by CODEML. Asterisks (*) indicate codons coding for putative antigen-binding sites that were defined according to Bjorkman et al. (1987). The dotted line indicates ω = 1. Error bars indicate the standard error of the mean. Arrows indicate significant positively selected sites identified by the Bayes Empirical Bayes procedure (*P* > 0.95).

Rates of nonsynonymous substitutions were higher than synonymous substitutions in the ABS in both the α1 and α2 domains and the non-ABS of the α1 domain (Fig. 5). Synonymous substitutions, however, were higher than nonsynonymous substitutions in the α3 domain (which has no ABS) and the non-ABS in the α2 domain. PAML models M2a (positive selection) and M8 (beta and ω), which permit positive selection at a subset of codon sites, gave a significantly better fit than did the models without positive selection in the α1 domain (Table 4). That is, positive selection signals were detected in the α1 domain. In the α2 domain, however, models that allow for positive selection did not provide a significantly better fit than the models of neutral evolution (Table 4). Parameter estimates (Table 4) indicate that 3.8% (under both M2a and M8) of α1 amino acid sites are under positive selection with ω = 13.7 (M2a) and ω = 14.4 (M8). Two sites were identified as being under positive selection (Table 4; Fig. 6); both PSS were within the ABS (Fig. 6; Kaufman et al. 1994).

**Table 4:** Positively selected sites (PSS), parameter estimates, log-likelihood values of different models of codon evolution, and summary of test statistics for the likelihood-ratio test (LRT) of the α1- and α2 domain of MHC class I genes in *Meles meles*

Domain	Model name		Log-likelihood	Parameter estimate(s)						PSS	LRT	Test statistic	*P* value
α1	M1a	nearly neutral	−588.31	*p*_0_ = 0.61	ω_0_ = 0.00	*p*_1_ = 0.39	ω_1_ = 1.00			Not allowed	M1a versus M2a	12.78	<0.002
	M2a	positive selection	−581.92	*p*_0_ = 0.51	ω_0_ = 0.00	*p*_1_ = 0.45	ω_1_ = 1.00	*p*_2_ = 0.04	ω_2_ = 13.74	**66N**, 67L			
	M7	beta	−588.32	*p* = 0.01	*q* = 0.01					Not allowed	M7 versus M8	12.74	<0.002
	M8	beta and ω	−581.95	*p*_0_ = 0.96	*p* = 0.01	*q* = 0.01	*p*_1_ = 0.04	ω = 14.36		**66N**, 67L			
α2	M1a	nearly neutral	−623.67	*p*_0_ = 0.66	ω_0_ = 0.00	*p*_1_ = 0.34	ω_1_ = 1.00			Not allowed	M1a versus M2a	0	1
	M2a	positive selection	−623.67	*p*_0_ = 0.66	ω_0_ = 0.00	*p*_1_ = 0.26	ω_1_ = 1.00	*p*_2_ = 0.08	ω_2_ = 1.00	*NS*			
	M7	beta	−623.79	*p* = 0.01	*q* = 0.03					Not allowed	M7 versus M8	0.24	0.89
	M8	beta and ω	−623.67	*p*_0_ = 0.66	*p* = 0.01	*q* = 70.51	*p*_1_ = 0.34	ω = 1.00		*NS*			

Log-likelihood values and estimated parameters for each model were calculated using CODEML in PAML 4.4b (Yang 2007). ω = *d_N_*/*d_S_* ratio; *p*_n_ is the proportion of amino acids in the ω_n_ site class. *p* and *q* are the statistical parameters of the beta distribution. PSS were identified in models M2a and M8 by Bayes Empirical Bayes (BEB) with posterior probabilities (PP) > 0.95 (Yang et al. 2005); sites with PP > 0.99 are shown in bold. *NS*, not significant. Test statistics = twice the difference of the log-likelihood ratios (2ΔInL). Degree of freedom is two for all LRTs. *P* value determined by comparing the test statistic to a *χ^2^* distribution.

One significant recombination event was detected in *M. meles* class I sequences, indicating that both Meme-MHC I*05 and Meme-MHC I*06 were likely to have originated from Meme-MHC I*PS08N and Meme-MHC I*04. The recombination signals were significant for RDP, GENECONV, BootScan, SiScan (all *P* < 0.001), MaxChi, and Chimera (both *P* < 0.01). Two recombination breakpoints were determined of which one was situated in exon 2 (nucleotide position 216 from the beginning of exon 2; MaxChi: *P* < 0.001) and one in intron 2 (nucleotide position 382; MaxChi: *P* < 0.001; Fig. 3).

### Phylogenetic analyses

**Figure 7:**
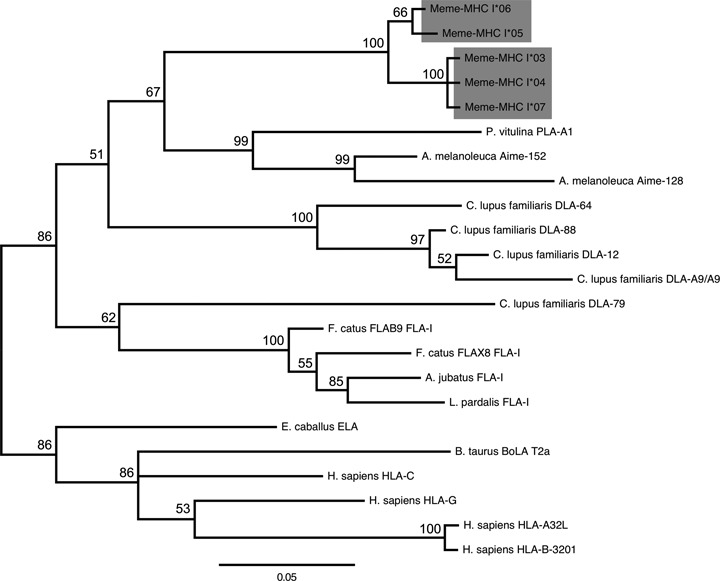
Phylogenetic tree of MHC class I exon 4/α3-domain sequences from *Meles meles* and other mammalian species (GenBank accession numbers are provided in the Materials and Methods), based on the 50% majority rule tree from the Bayesian analysis. Bayesian posterior probabilities above 50% are shown above the branches. *Meles meles* class I sequences are marked with *gray boxes*. *PS* signifies pseudogenes with a frameshift. *N* signifies the presence of genomic sequences, where no cDNA sequences were found.

The phylogenetic tree of α1 (Fig. 4A) highlights that sequences from *M. meles* did not form a monophyletic clade, rather they were intermingled with phocine, canine, and ursine sequences. No sequences from the other species included in the analysis formed a monophyletic clade, except for those from *H. sapiens* and *M. schauinslandi*. But the support of clades with mixed sequences from difference species was not strong (e.g., bootstrap value = 56 for the clustering of *Aime*-128 and Meme-MHC I*01, 02, and *04*). Some *M. meles* pseudogenes, however, formed highly or moderately supported clades without grouping with any putatively functional sequences (e.g., Meme-MHC I*PS02 and Meme-MHC I*PS03; Meme-MHC I*PS08N, Meme-MHC I*09N, and Meme-MHC I*PS10). For the sequences encoding the α2 domain (Fig. 4B), all *M. meles* sequences, except for Meme-MHC I*PS08N, formed a monophyletic clade. Within this clade, pseudogenes Meme-MHC I*PS04N, Meme-MHC I*PS02, and Meme-MHC I*PS03 grouped together. Sequences from *A. melanoleuca* and *H. sapiens* also formed monophyletic clades. For α3 sequences, *M. meles* formed a distinct and highly supported clade (posterior probability [PP] support = 1.0; Fig. 7) that grouped together with *P. vitulina* and *A. melanoleuca* (PP = 0.67). This clade further grouped with all carnivores in our comparison including canine and feline sequences (PP = 0.86), separating it from equine, bovine, and human sequences.

## Discussion

### Diversity and transcription of MHC class I sequences

This is the first study to characterize MHC class I genes in *M. meles*. Moreover, this work was performed using both the genome and transcriptome. The sequences identified included pseudogenes and putatively functional sequences that encode domains necessary to form functional class I molecules. The major structural features (Kaufman et al. 1994) that distinguish class Ia molecules are all present in these putatively functional sequences, including highly conserved amino acid residues that bind the N- and C-termini of the peptide, cysteine (C) residues that form the disulphide bonds, an N-linked glycosylation site, a threonine (T) residue for interaction with TAP complex, and regions to interact with CD8 glycoproteins on the T cells. In addition, we demonstrate that all putatively functional sequences were expressed in the RNA level in whole blood. These features, together with the detected polymorphisms, indicate that these sequences belong to at least two class Ia loci, in contrast to class Ib genes that are typically monomorphic (Rodgers and Cook 2005).

The variability in the number of class I sequences in *M. meles* is intermediate compared to other carnivores. The most closely related species for which class I genes have been characterized is the Hawaiian monk seal (*M. schauinslandi*; Aldridge et al. 2006), which has at least two loci. No variability was found, however, within more than 80 individuals of this endangered seal species. Within the infraorder Arctoidea, the giant panda (*A. melanoleuca*) has two classical genes (*Aime*-152 and *Aime*-128; Pan et al. 2008), plus a nonclassical gene closely related to DLA-79. *Aime*-152 appears to be monomorphic, while nine *Aime*-128 sequences were found in five individuals (Pan et al. 2008). The carnivores that, to date, have had their MHC organization characterized most thoroughly are *C. lupus familiaris* and *F. catus*. In the feline leukocyte antigen (FLA), there are at least three class Ia genes, in common with human and murine MHCs (Yuhki et al. 2007). In DLA (Burnett et al. 1997; Kennedy et al. 1999; Wagner et al. 1999; Wagner 2003), there is one class Ia gene (DLA-88), three class Ib genes (DLA-79, -12, and -64), and two pseudogenes (DLA-53 and -12a). Among these, DLA-88 is the most polymorphic gene, with more than 50 alleles identified (Wagner 2003). As discussed above, the number of class I genes and their organization can differ between species greatly (Kelley et al. 2005). Mammals usually possess 1−3 class I genes (Nei et al. 1997) and have a variable range of polymorphism (Parham and Ohta 1996; Wagner 2003). Considering the extensive geographical range of *M. meles* and their considerable socio-spatial variability (Macdonald et al. 2004; Rosalino et al. 2004; Newman et al. 2011), it is highly likley that more sequences will be detected as and when other populations are examined.

Our transcription analysis demonstrated that not all the detected sequences were expressed in whole blood. In addition to the seven putatively functional sequences, for which the sequences detected from gDNA and cDNA were identical; nine sequences were identified as pseudogenes. The phylogenetic analyses indicate that Meme-MHC I*PS02, Meme-MHC I*PS03, and Meme-MHC I*PS04N, which form a strongly supported clade, belong to the same pseudogene locus, with Meme-MHC I*PS02 and Meme-MHC I*PS03 both present in two individuals and Meme-MHC I*PS04N detected in another two individuals. Another grouping of Meme-MHC I*PS08N, Meme-MHC I*09N, and Meme-MHC I*PS10 indicated that these belong to two closely related pseudogene loci, as 1−3 sequences of this group were detected from seven individuals separately. Other studies have shown that many MHC sequences can be detected at the genomic level, but not at the cDNA level (de Groot et al. 2004). The presence of expressed nonfunctional MHC pseudogenes has also been reported (Mayer et al. 1993; Fernandez-Soria et al. 1998), even in the class II genes of *M. meles* (Sin et al. 2012). This is concordant with the finding that the MHC class I and class II regions have duplicated many times, generating many pseudogenes in addition to novel functional genes (Beck et al. 1999).

### Evolution of the MHC class I genes

Our phylogenetic analyses reveal the evolutionary histories of the class I domains we examined to be very different from each other. Within the α1 domain, there were more nonsynonymous than synonymous nucleotide substitutions in both the ABS and non-ABS, contributing to a higher nucleotide and amino acid sequence diversity. Evidence of positive selection was detected in the α1 domain, in which PSS were all within the ABS (Fig. 6A). *Meles meles* sequences of the α1 domain were not monophyletic within this species but intermingled with sequences from other species (e.g., *P. vitulina*); a phenomenon characteristic of MHC genes. This could be due to trans-species polymorphism (Klein 1987; Hughes and Yeager 1998; Klein et al. 1998), whereby balancing selection maintains this ancestral variation over a long period of time (many generations), even after species divergence (Penn and Potts 1999; Bernatchez and Landry 2003), leading to differences between gene tree and species tree. As the α1 domain is responsible for antigen binding, the diversifying and balancing selection that drives and maintains this ABS polymorphism permits a population to present a wider repertoire of antigens, thus increasing its ability to combat pathogenic and parasitic infections (Hughes and Nei 1992; Hughes and Yeager 1998). The clustering of sequences among species could also be due to orthology, whereby sequences from an orthologous gene will cluster together, which may produce a similar clustering pattern as that observed with trans-species polymorphism. Without the availability of information about loci identity, it was not possible to disentangle between signals for orthology and trans-species polymorphism. Recombination between exons that encode ABSs can also increase allelic diversity in MHC genes (Ohta 1991; Jakobsen et al. 1998; Shum et al. 2001; Bos and Waldman 2006). We detected a recombination event at the 3′ end of exon 2 that may have increased variation in *M. meles* class I genes. Nevertheless, point mutation and selection have been proposed as the major reason for high diversity in the MHC (Nei et al. 1997; Piontkivska and Nei 2003; Nei and Rooney 2005).

As is the case for α1 domain, the α2 domain functions as an antigen-binding domain and showed a higher nonsynonymous/synonymous rate ratio (ω) within the ABS than in the non-ABS (Fig. 6B). Comparisons of maximum likelihood models, which allow for positive selection (M2a and M8) relative to their corresponding null models, detected no significant positive selection acting on this region, however. A possible explanation for less intense diversifying selection on the α2 domain would be that it has more conserved sites than the α1 domain and so purifying selection may act to maintain the structural features of the MHC molecule. Our phylogenetic analyses also showed that all *M. meles*α2 domain sequences were grouped together except for one pseudogene (Meme-MHC I*PS08N clustering with DLA-64 but with low bootstrap support [72.4]; Fig. 4B); a very different pattern to the pattern that we observed in the α1 domain. The birth-and-death model of evolution predicts that this type of clustering by species (Nei et al. 1997; Piontkivska and Nei 2003) will result in less orthologous relationships, due to higher rates of gene duplication and deletion. Mechanistically, a differential rate of birth-and-death evolution explains the more rapid turnover rate of class I genes than class II genes in mammals (Hughes and Nei 1989; Piontkivska and Nei 2003) and possibly reveals why sequences are more closely related within *M. meles* than to sequences from other species. The α1 sequences we observed, however, showed that ancestral polymorphism or orthology at multiple lineages was maintained after the analyzed species (Fig. 4A) diverged from one another, which indicates that different selective pressures act on different class I gene regions. Under this birth-and-death model, balancing selection at the α1 domain should maintain ancestral polymorphism even after species divergence, while strong purifying selection and recent duplication are required to produce a species-specific clustering at the α2 domain.

Alternatively, and more plausibly, species-specific gene clusters may result from concerted evolution (Hess and Edwards 2002; Joly and Rouillon 2006), where genes are homogenized by recombination. Recombination in the α2 coding region could produce *M. meles* sequences that are more similar to one another than to the sequences from other species. Under this scenario, a recombination breakpoint is needed to separate selection on the α1 and α2 regions, given that they exhibit different evolutionary histories. Our recombination analysis exposed that there are indeed recombination breakpoints located in the middle of the α-helix of the α1 domain and in the middle of intron 2, which is between the α1 and α2 coding exons. These recombination breakpoints indicate the probable crossover location and they most likely contributed to the independent evolutionary histories of the α1 and α2 domains by recombination in the α2 encoding region. Recombination within intron 2 has also been reported in other species (Holmes and Parham 1985; Shum et al. 2001; Bos and Waldman 2006), which gives greater support to the hypothesis of concerted evolution through shuffling of the α1 and α2 domains. These recombination breakpoints are shared among some *M. meles* sequences, demonstrating that breakpoints are not random and that these recombination events are due to in vivo recombination but not in vitro chimera formation. Coincidentally, the degenerate 13-bp sequence motif, which is crucial in the crossover event of human recombination hotspots (Myers et al. 2008), was also found in the *M. meles* intron 2 sequences in which the recombination breakpoints were identified (Fig. 3). Variation in the zinc-finger protein PRDM9, however, affects species-specific binding to the sequence motif (Myers et al. 2010); hence, more studies are needed to elucidate the recombination mechanism in *M. meles*.

Phylogenetic relationships of the α3 encoding sequences were more similar to that of the α2 than the α1 domain, demonstrating species-specific clustering of *M. meles*α3 sequences. This indicates that a recombination event most plausibly separated the α1 domain and the remaining 3′ segments of the gene, leading to inconsistencies between the α1 gene tree and the α2 and α3 gene trees. However, the α3 domain is not antigen binding and it shows a very low number of mutations and polymorphic amino acid residues. Compared with the antigen-binding domains that are under positive selection, which increases diversity, strong purifying selection could eliminate mutations leading to unfavorable changes in the molecular structure or the regions that interact with T cells (Kaufman et al. 1994). The relatively conserved α3 domain is thus used to reconstruct evolutionary relationships more frequently than the α1 and α2 domains (e.g., Glaberman and Caccone 2008). Here, the phylogenetic relationships of the α3 sequences follow the molecular phylogeny of the extant Carnivora (Flynn et al. 2005; Fulton and Strobeck 2006), in which Musteloidea, Pinnipedia, and Ursoidea together form the infraorder Arctoidea. This infraorder then groups with Canidae to form the suborder Caniformia and together with Feliformia comprise the order Carnivora. An exception is *P. vitulina* that forms a clade with *A. melanoleuca* instead of *M. meles*. Musteloidea diverged from Ursoidea and Pinnipedia around 36 and 35.5 million years ago, respectively (Bininda-Emonds et al. 1999). The proximity of these divergence times might lead to incomplete lineage sorting (Maddison and Knowle 2006), which is common in highly polymorphic genes (Lu 2001).

## Conclusions

Our findings highlight the importance of examining gene regions separately in order to obtain a more comprehensive understanding of MHC evolution. While the MHC class II genes (e.g., *DRB* and *DQB*; Sin et al. 2012) in *M. meles* did not exhibit extensive trans-species polymorphism, it nevertheless was observed. The class II genes are therefore likely to be undergoing balancing selection, whereas phylogenetic inconsistency between the α1 and α2/α3 domains of the class I genes indicates that these domains have different evolutionary histories. Concerted evolution provides a more plausible hypothesis than birth-and-death evolution in the α2 and α3 domains, given the intron 2 breakpoint and separate evolutionary history of the α1 domain. Probable recombination has homogenized the α2 and α3 encoding exons, after ancestral gene duplication. As the α2 domain is antigen binding, whereas α3 is not, these domains were subject to different selective pressures leading to the observed difference in the degree of sequence divergence over evolutionary time. Separated from the α2 and α3 encoding exons, the α1 domain is likely to be subject to balancing selection (Nei et al. 1997). The high number of pseudogenes we found is typical in class I loci, which often show a high turnover rate, generating an abundant number of pseudogenes with various degrees of divergence from their functional counterparts (Hughes 1995; Cadavid et al. 1996; Beck et al. 1999; Piontkivska and Nei 2003). Nonfunctional sequences could serve as a reservoir for genetic exchange (Ohta 1991; de Groot et al. 2004), which would be the case here if the recombination event happened after the parental sequence Meme-MHC I*PS08N lost its function.

This is the first study to characterize MHC class I genes in a species within the superfamily Musteloidea. Given the polygynandrous mating system of *M. meles*, where high levels of extra-group paternity are observed (Dugdale et al. 2007), further studies of mate choice and MHC would be informative. Examination of the association between MHC genotypes and pathogens could also have significant implications for research into bTB epidemiology in badgers (Allen et al. 2010).

## Supporting Information

**Table S1.** Different *Meles meles* MHC class Ialleles could be obtaining by PCR amplification using the primersmarked with *ticks.*
