# Supplementary material for: Evolution of MHC class I genes in the European badger (Meles meles)
Source: Ecol Evol. 2012 Jul;2(7):1644–62. doi: 10.1002/ece3.285 (PMC3434948; doi:10.1002/ece3.285)
Supplement: Supplementary file 1 [file ece30002-1644-SD1.pdf]

Evolution of MHC class I genes in the European badger (*Meles meles*)

YUNG WA SIN*†, HANNAH L. DUGDALE†‡§, CHRIS NEWMAN*, DAVID W. MACDONALD* & TERRY BURKE†

**Wildlife Conservation Research Unit, Department of Zoology, University of Oxford, Recanati-Kaplan Centre, Tubney House, Abingdon Road, Tubney, Abingdon, Oxfordshire OX13 5QL, UK*

†*NERC Biomolecular Analysis Facility, Department of Animal and Plant Sciences, University of Sheffield, Western Bank, Sheffield, South Yorkshire, S10 2TN, UK*

‡*Behavioural Ecology and Self-Organization, University of Groningen, PO Box 11103, 9700 CC Groningen, Netherlands*

§*Theoretical Biology, University of Groningen, PO Box 11103, 9700 CC Groningen, Netherlands*

Corresponding author (Y. W. Sin) e-mail address: yungwa.sin@zoo.ox.ac.uk

Published in: Ecology and Evolution

Supplementary material

Table S1 Different *M. meles* MHC class I alleles could be obtained by PCR amplification using the primers marked with *ticks*. Primer information and amplified region are given in Table 1 and Figure 1

	Primers					
	Meme-MHCIex1F	Meme-MHCIex2F	PpLAa1L250	Meme-MHCIex3F	Meme-MHCIex3R	Meme-MHCIex6R
*Meme-MHC I*01*		✔		✔	✔	
*Meme-MHC I*02*		✔		✔	✔	
*Meme-MHC I*03*	✔	✔	✔	✔	✔	✔
*Meme-MHC I*04*	✔	✔	✔	✔	✔	✔
*Meme-MHC I*05*	✔	✔	✔	✔	✔	✔
*Meme-MHC I*06*	✔	✔		✔	✔	✔
*Meme-MHC I*07*	✔	✔		✔	✔	✔
*Meme-MHC I*PS08N*		✔		✔	✔	
*Meme-MHC I*09N*		✔	✔			
*Meme-MHC I*PS10*		✔	✔			
*Meme-MHC I*11*		✔	✔			
*Meme-MHC I*PS12N*		✔	✔			
*Meme-MHC I*PS01*	✔					✔
*Meme-MHC I*PS02*	✔					✔
*Meme-MHC I*PS03*	✔					✔
*Meme-MHC I*PS04N*				✔	✔	
